# Lectures on Surgical Pathology, Delivered at the Royal College of Surgeons of England

**Published:** 1865-04

**Authors:** 


### Book Notices.

LECTURES ON SURGICAL PATHOLOGY, delivered at the Royal College of Surgeons of England. By JAMES PAGET, F.R.S., Surgeon Extraordinary to her Majesty the Queen, &c., &c. Revised and edited by WILLIAM TURNER, M.B., Lond., F.R.C.S.E., F.R.S.E., &c. Third American Edition. Philadelphia: LINDSAY & BLAKISTON. 1865.

This is a full-sized octavo volume of 737 pages, printed on good paper, fair type, and bound in cloth. It is a new edition of a very valuable work, but which has been so long before the profession, that its merits are already extensively appreciated. It contains thirty-six lectures on the general subjects of nutrition, healthy and morbid; reparation of injuries, wounds, &c.; inflammation, its products and consequences; tumors and morbid growths; and tubercle. The work is illustrated by 117 woodcuts. It is a work worthy of the careful study of every physician and surgeon.

For sale by S. C. GRIGGS & Co., 41 Lake Street, Chicago.

